# Clinicopathological Relationships in an Aged Case of DOORS Syndrome With a p.Arg506X Mutation in the *ATP6V1B2* Gene

**DOI:** 10.3389/fneur.2020.00767

**Published:** 2020-08-07

**Authors:** Dénes Zádori, Levente Szalárdy, Zita Reisz, Gabor G. Kovacs, Rita Maszlag-Török, Norbert F. Ajeawung, László Vécsei, Philippe M. Campeau, Péter Klivényi

**Affiliations:** ^1^Department of Neurology, Interdisciplinary Excellence Center, Faculty of Medicine, Albert Szent-Györgyi Clinical Center, University of Szeged, Szeged, Hungary; ^2^Department of Pathology, Faculty of Medicine, Albert Szent-Györgyi Clinical Center, University of Szeged, Szeged, Hungary; ^3^Institute of Neurology, Medical University of Vienna, Vienna, Austria; ^4^Department of Laboratory Medicine and Pathobiology, Tanz Centre for Research in Neurodegenerative Disease, University of Toronto, Toronto, ON, Canada; ^5^Laboratory Medicine Program & Krembil Brain Institute, University Health Network, Toronto, ON, Canada; ^6^CHU Sainte-Justine Research Center, Université de Montréal, Montreal, QC, Canada; ^7^MTA-SZTE Neuroscience Research Group, University of Szeged, Szeged, Hungary; ^8^Department of Pediatrics, Sainte-Justine University Hospital Center, Montreal, QC, Canada

**Keywords:** DOORS syndrome, *ATP6V1B2* gene, lysosome, neuropathology, tauopathy

## Abstract

DOORS [deafness, onychodystrophy, osteodystrophy, intellectual disability (mental retardation), and seizures] syndrome can be caused by mutations in the *TBC1D24* and *ATP6V1B2* genes, both of which are involved in endolysosomal function. Because of its extreme rarity, to date, no detailed neuropathological assessment has been performed to establish clinicopathological relationships and, thereby, understand better the neurobiology of this disease in aged cases. Accordingly, the aim of the current study was to highlight the clinicopathological characteristics of a novel case with a presumable *de novo* mutation in the *ATP6V1B2* gene from a neuropathological point of view. This Caucasian male patient, who died at the age of 72 years, presented all the typical cardinal signs of DOORS syndrome. In addition, behavioral alterations, pyramidal signs, and Parkinsonism were observed. The p.R506X pathogenic mutation identified in the *ATP6V1B2* gene was responsible for the clinical phenotype. The detailed neuropathological assessment revealed a limbic-predominant tauopathy in the forms of argyrophilic grain disease, primary age-related tauopathy, and age-related tau-astrogliopathy. In summary, we present the first detailed clinicopathological report of a patient with DOORS syndrome harboring a pathogenic mutation in the *ATP6V1B2* gene. The demonstrated tauopathy may be considered as a consequence of lysosomal and/or mitochondrial dysfunction, similar to that found in Niemann–Pick type C disease, which is another lysosomal disorder characterized by premature neurodegenerative disorder.

## Introduction

DOORS syndrome is an extremely rare condition that is characterized by the combination of the core clinical features of deafness, onychodystrophy, osteodystrophy, intellectual disability (mental retardation), and seizures, in addition to specific craniofacial anomalies (1). The most frequently identified genetic cause of DOORS syndrome is homozygous mutation in the *TBC1D24* gene (2). However, overlapping syndromes exist, including DDOD (dominant deafness and onychodystrophy), with similar, albeit less severe, symptoms and signs (3). The first report of another possible candidate gene, *ATP6V1B2* (p.Arg506X *de novo* heterozygous mutation), in DDOD syndrome was published in 2014 (4). To date, a mere 27 cases of DDOD have been reported (5, 6), four of which have genetic confirmation (4, 6). Furthermore, two patients harboring a p.R485P heterozygous mutation were confirmed to have Zimmermann–Laband syndrome (7). Interestingly, *ATP6V1B2* gene mutations have also been identified in some patients presenting with complete DOORS syndrome (unpublished findings), or in clinical syndromes overlapping with the three entities mentioned above in patients harboring the p.E374Q or p.L398V mutations in a heterozygous state (8, 9), thus widening the clinical picture of these syndromes (Table 1). Although it has been revealed that the p.Arg506X mutation may impair lysosome acidification in the brain (4, 10), there are no detailed neuropathological reports of the clinicopathological relationships associated with this mutation. Accordingly, the aim of the current study was to provide a detailed clinicopathological assessment of a novel case with a presumable *de novo* mutation in the *ATP6V1B2* gene, in accordance with the CARE (CAse REport) guidelines (11), with special focus on the delineation of the neuropathological signs of different tauopathies.

**Table 1:** Clinical characteristics of genetically identified cases with mutation in the *ATP6V1B2* gene.

	**Our patient**	**Yuan et al. (4)**	**Kortüm et al. (7)**	**Menendez et al. (6)**	**Popp et al. (8)**	**Shaw et al. (9)**
No. of affected individuals	1	3	2	1	1	7
Diagnosis	DOORS	DDOD	ZLS	DDOD	ID with hypotonia and epilepsy	ZLS-like disease
Mutation	p.R506X	p.R506X	p.R485P	p.R506X	p.E374Q	p.L398V
Inheritance	*De novo*	*De novo*	*De novo*	*De novo*	*De novo*	Autosomal dominant
Deafness	1/1	3/3	1/2	1/1	–	–
Absent/hypoplastic finger or toe nails	1/1	3/3	2/2	1/1	–	–
Thumbs	Triphalangeal and triphalangeal-like (1/1)	Finger-like (3/3)	Long, finger-like (1/2)	Triphalangeal (1/1)	Normal (1/1)	Normal (7/7)
Brachydactyly	1/1	3/3	2/2	1/1	–	–
Scoliosis	–	NR	1/2	–	NR	–
Craniofacial dysmorphism	1/1	–	2/2	–	NR	4/7
ID	1/1	–	2/2	–	1/1	4/7
Seizure	1/1	–	–	–	1/1	7/7

*DDOD, dominant deafness and onychodystrophy; DOORS, deafness, onychodystrophy, osteodystrophy, mental retardation, and seizures; ID, intellectual disability; NR, not reported; ZLS, Zimmermann–Laband syndrome*.

## Case Report

### Case History and Neurological and Related Alterations

The Caucasian male patient reported here was born at term and his post-natal period was uneventful. Although the available data are limited, in addition to deafness, intellectual disability, and structural abnormalities (as detailed later), no other major alteration was reported with regard to his early childhood. He was not able to attend school, lived with his parents, and needed some assistance in daily activities in his later life. His family history was negative for the condition described above and no consanguinity was reported in the family. His medical history was relatively uneventful until the age of 52, when he was admitted to the neurological department after two generalized tonic–clonic seizures. No provoking factors could be identified. Subsequently, he was free of seizures until the age of 55, when he was admitted again to the neurological department after two additional generalized tonic–clonic seizures. Although electroencephalography (EEG) did not find any abnormalities, a diagnosis of epilepsy was established based on the presence of repeated unprovoked seizures, and carbamazepine treatment was initiated accordingly. Thereafter, only one seizure was reported. At the age of 63, resting tremor was identified during a regular check-up. At the age of 65, he was admitted to the psychiatric unit because of impulsive behavior and self-harm. Clonazepam and risperidone were initiated, providing appropriate symptom control. Between the ages of 65 and 70, he was admitted several times to the surgical unit because of incomplete or complete ileus events, which were managed conservatively, with the exception of one occasion. At the age of 66, he was admitted to the Department of Internal Medicine with the suspicion of slight cardiac decompensation. Echocardiography demonstrated a dilated aortic root, mitral prolapse, and minimal pericardial fluid, but an overall good myocardial function. The leg edema responded well to diuretics. At the age of 67, carbamazepine was changed to valproic acid without any known reason (no medical record is available regarding this switch). At the age of 69, he was referred to the emergency department with a general malaise. The patient was admitted to the neurological department for a diagnostic workup of his unknown syndrome.

**Figure 1:**
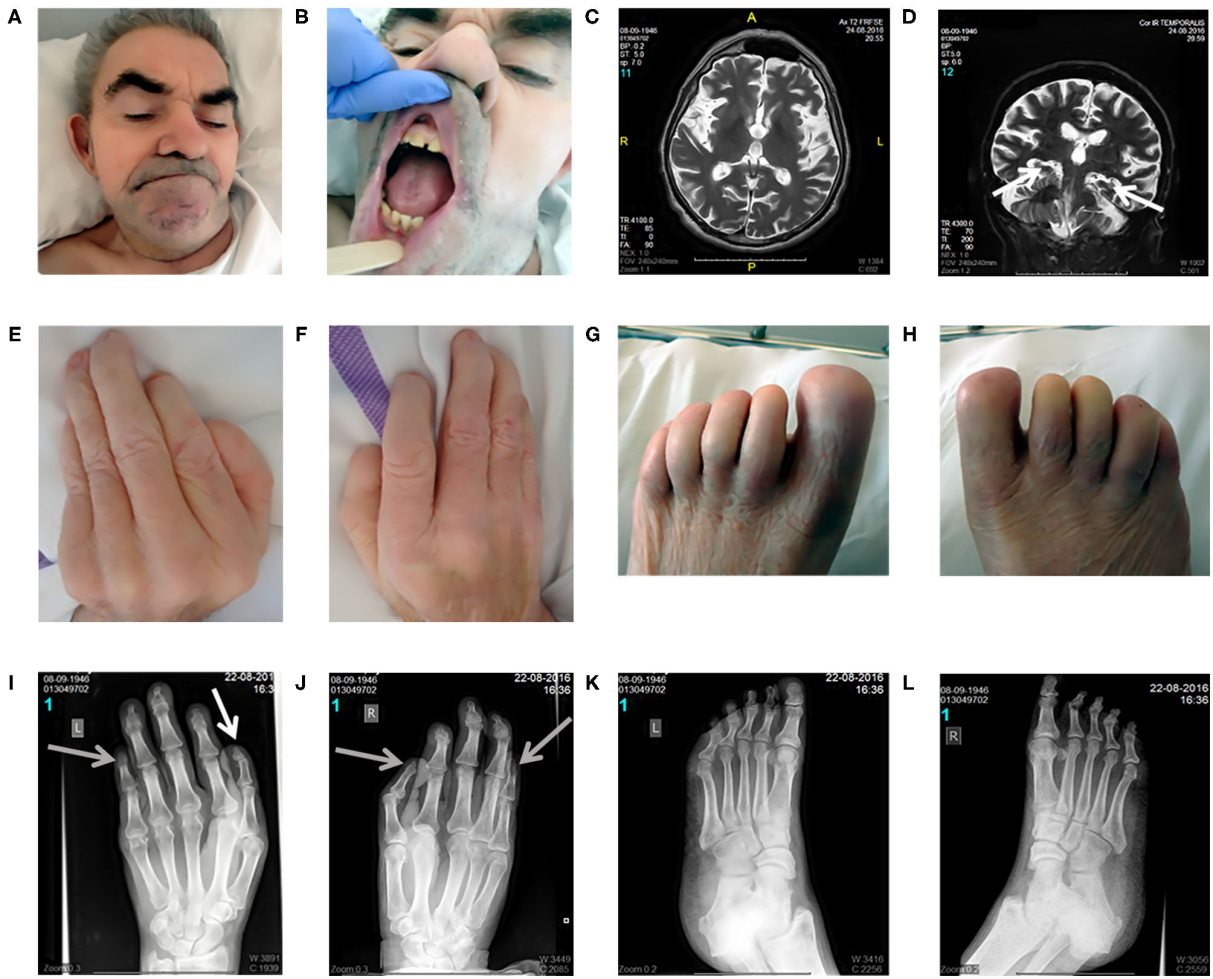
Clinical and radiomorphological characteristics of an aged case of DOORS syndrome. Craniofacial dysmorphism (coarse facies, hypertrichosis, broad nasal bridge, wide mouth, smooth philtrum, slightly hypertrophic gingiva, widely spaced teeth, and large ears) could be observed distinctively **(A,B)**. Brain MRI revealed mild generalized supratentorial and infratentorial atrophy, predominantly affecting the temporal lobes, including moderate hippocampal atrophy [white arrows; **(C,D)**]. Regarding limb alterations, onychodystrophy, and abnormal fingers and toes with small distal phalanges were detected **(E–H)**. Accordingly, the X-ray assessment demonstrated the presence of a triphalangeal left thumb [white arrow; **(I)**] and a triphalangeal-like right thumb, but with suspected synostosis of the distal phalanges. A similar synostosis of the distal phalanges could be observed in the fifth fingers [gray arrows indicate these three synostosis; **(I,J)**]. Regarding the feet, the middle phalanges could not be distinguished and the distal phalanges of the first toes were smaller than normal **(K,L)**. Written informed consent was obtained from the legal guardian of the participant in the study for the publication of photo materials with identifying information.

On inspection, craniofacial dysmorphism (coarse facies, hypertrichosis, broad nasal bridge, wide mouth, smooth philtrum, slightly hypertrophic gingiva, widely spaced teeth, and large ears; Figures 1A,B), onychodystrophy, and abnormal fingers and toes with small distal phalanges (Figures 1E–H) could be observed. On neurological examination (Supplementary Material 1), in addition to the well-known deafness, he presented with pyramidal signs, including latent left-sided hemiparesis with slight tetraspasticity (with left-sided predominance), the presence of Wernicke–Mann posture, brisk radial reflexes with clonus on the right side, inverted radial reflex and Hoffmann's sign bilaterally, and left-sided Babinski sign with fanning of the toes. Furthermore, signs of Parkinsonism including rigidity with left-sided predominance, moderate symmetrical hypokinesia, lip tremor, hypomimia, and occasional upper and lower limb resting tremor with slight action component predominantly on the left side could also be observed (bradykinesia could not be tested because of the lack of appropriate cooperation). Unaided stance and gait could not be achieved. Finally, because of the lack of appropriate cooperation, a better characterization of his intellectual disability was not possible and the age-related cognitive decline as a part of the mental dysfunction could not be estimated.

The patient died at the age of 72 years because of respiratory insufficiency caused by aspiration.

### Imaging Studies

X-ray images of the limbs (Figures 1I–L) demonstrated a triphalangeal left thumb, and a triphalangeal-like right thumb, albeit with suspected synostosis of the distal phalanges. A similar synostosis of the distal phalanges could be observed in the fifth fingers. Regarding the feet, the middle phalanges could not be distinguished, and the distal phalanges of the first toes were smaller than normal.

Brain MRI revealed mild generalized supratentorial and infratentorial atrophy predominantly affecting the temporal lobes, including a moderate hippocampal atrophy (Figures 1C,D). Brain Tc-99 m single-photon emission computed tomography (SPECT) performed at the age of 63 demonstrated a globally reduced cortical vascular reserve capacity with the involvement of basal ganglia, in addition to brain atrophy.

### Neuropathological Assessment

#### Macroscopic Neuropathology

On macroscopic neuropathological evaluation, the external examination revealed frontoparietal and parasagittal thickening and opacity of the dura. The circle of Willis and the cranial nerves were intact. The cortical ribbon and the sulci were mostly retained, but a mild atrophy of the temporal lobes could be observed. On coronal sections, a mild thinning of the temporal gyri was accompanied by moderate symmetrical, bilateral hippocampal atrophy (Supplementary Material 2A). Accordingly, the lateral sulci and the temporal horns of the lateral ventricles were moderately dilated, in line with the MRI findings. Despite this regional atrophy, the total brain weight was well-preserved, at 1,460 g. Small lacunes were detected both in the putamen and in the surrounding deep white matter, whereas the caudate and the thalamic nuclei were unaffected (Supplementary Material 2B). The cerebellum and the brainstem did not show any macroscopic alterations.

#### Microscopic Neuropathology

##### Light Microscopy

The midfrontal and cingulate cortices exhibited relatively preserved neurons without reactive gliosis. There was a lack of vascular lesions or inflammatory infiltrates. Immunostaining for amyloid-beta (Aβ) did not show parenchymal or vascular deposits (image not shown). Immunostaining for phospho-tau (AT8) revealed few neurons with diffuse fine granular immunoreactivity, similar to pretangles, and occasionally with small fibrillar conglomerates, similar to early neurofibrillary tangles (NFTs). Small amounts of neuropil threads were also detected. In the cingulate, there were no ballooned neurons and grain-like tau positivity was observed only occasionally. In the temporal cortex, a more pronounced neuronal tau pathology was detected and that was accompanied occasionally by granular/fuzzy astrocytes in the gray matter and several oligodendroglial coiled bodies in the white matter. A few grain-like structures were also observed. The parietal cortex showed a single neuronal tau pathology, whereas the occipital cortex did not show any tau pathology. No Aβ deposition was detected in these regions.

**Figure 2:**
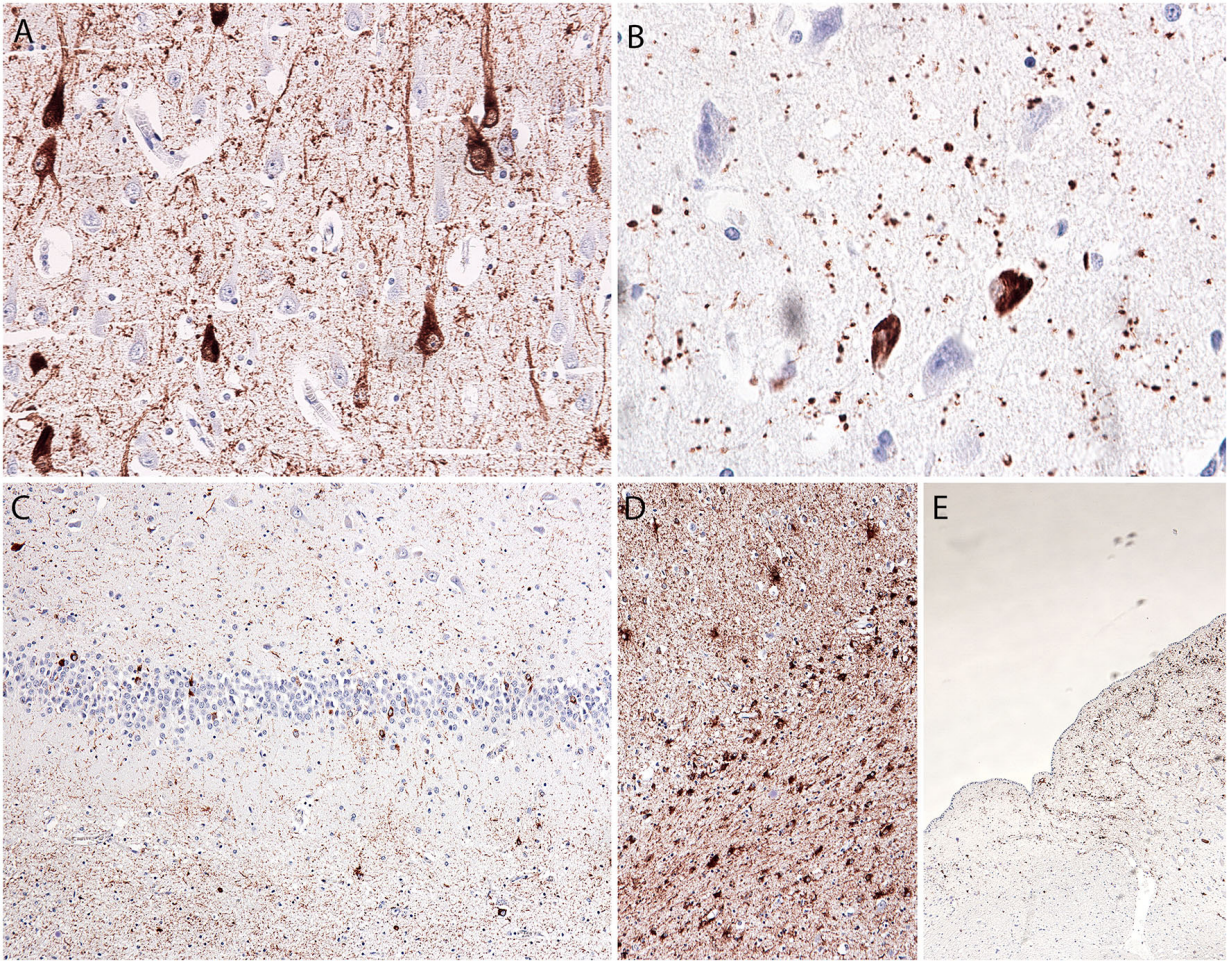
Microscopic *postmortem* neuropathological alterations of an aged patient with DOORS syndrome. Neurofibrillary tangles, pretangles, neuropil threads, and grains in the hippocampal CA1 area [AT8; **(A)**]. Tangles and grains in the transentorhinal cortex [p62; **(B)**]. Tangles and pretangles in the dentate gyrus with oligodendroglial coiled bodies in the white matter [bottom part; AT8; **(C)**]. Age-related tau-astrogliopathy (ARTAG) in the amygdala with granular-fuzzy astrocytes (upper part) and thorn-shaped astrocytes [lower part; AT8; **(D)**]. ARTAG in the medulla oblongata with thorn-shaped astrocytes [AT8; **(E)**].

The hippocampus and amygdala showed a prominent tau pathology in the form of NFTs (CA1 and entorhinal cortex), pretangles (all hippocampal subregions, dentate gyrus, entorhinal cortex, and inferior temporal gyrus), and neuropil threads and grains (p62-immunopositive; mostly in the entorhinal cortex, CA1, and amygdala), as well as granular/fuzzy astrocytes in the gray matter (highest density in the amygdala), thorn-shaped astrocytes in the white matter in the subependymal (inferior horn of the lateral ventricle) and subpial areas, and oligodendroglial coiled bodies and threads in the white matter (Figures 2A–E). The amygdala showed reactive astrogliosis, without prominent accumulation of ballooned neurons. There were no signs of hippocampal sclerosis. Immunostaining for the phosphorylated TAR DNA-binding protein 43 (phospho-TDP-43) and α-synuclein was negative (images not shown).

Despite the presence of a reduced vascular reserve capacity demonstrated by SPECT, the basal ganglia, and thalamus did not exhibit prominent vascular lesions apart from widened perivascular spaces; furthermore, there was a lack of inflammatory infiltrations. In the nucleus accumbens, several pretangles, and threads were observed, but no grains. Thorn-shaped astrocytes accumulated in the frontobasal white matter. The basal nucleus of Meynert showed a few NFTs.

In the brainstem and cerebellum, the anatomical structures were well-preserved and there was no significant neuronal loss or vascular lesions. With the exception of a few NFTs and pretangles in the locus coeruleus, there was no tau pathology or α-synuclein pathology. The regional distribution of tau pathology is presented in Table 2.

**Table 2:** Distribution of brain tau pathology in an aged case of DOORS syndrome with mutation in the *ATP6V1B2* gene.

**Brain region**	**Grade**
Midfrontal cx	+
Temporal cx	+++
Parietal cx	+
Occipital cx	0
Cingulate cx	+
Entorhinal cx	+++
Frontobasal white matter	+
Hippocampus (CA1)	+++
Dentate gyrus	+++
Amygdala	+++
Meynert nucleus	+
Nucleus accumbens	++
Basal ganglia	0
Thalamus	0
Cerebellum	0
Subependymal white matter including that of brainstem	++
Locus coeruleus	+
Other brainstem	0

*cx, cortex; DOORS, deafness, onychodystrophy, osteodystrophy, mental retardation, and seizures*.

***Method used for immunohistochemistry**.* Formalin-fixed, paraffin-embedded tissue blocks (2.5 × 2.0 cm) were applied. The presence of astrogliosis/microgliosis/neuronal loss and the degree of deposition of various proteins were semi-quantitatively (none, mild, moderate, and severe) evaluated in the following anatomical regions: frontal, temporal, parietal, occipital, and premotor cortices; basal ganglia; cerebellum; thalamus; amygdala; hippocampus; and the mesencephalon, pons, and medulla oblongata in the brainstem. In addition to Hematoxylin & Eosin and Luxol Fast Red staining, the following monoclonal antibodies were used for immunohistochemistry: anti-phospho-tau AT8 (pS202/pT205, 1:200; Pierce Biotechnology, Rockford, IL, USA), anti-phospho-TDP-43 (pS409/410, 1:2,000, Cosmo Bio, Tokyo, Japan), anti-α-synuclein (1:2,000, clone 5G4; Roboscreen, Leipzig, Germany), anti-Aβ (1:50, clone 6F/3D; Dako, Glostrup, Denmark), and anti-p62 (1:1,000; BD Transduction, Lexington KY, USA). The Dako EnVision Detection System, Peroxidase/DAB, Rabbit/Mouse (Dako, Glostrup, Denmark), was used for the visualization of antibody reactions.

##### Electron microscopy

Although the postmortem delay resulted in a considerable decrease in sample quality for transmission electron microscopy (TEM), the images obtained demonstrated a relatively preserved mitochondrial (Supplementary Material 3A) and synaptic vesicle structure (Supplementary Material 3B). However, the morphology and numbers of lysosomes and phagosomes could not be assessed with appropriate quality.

***Method used for electron microscopy**.* For ultrastructural examination, tissue specimens were fixed with 2% glutaraldehyde in phosphate-buffered saline containing 2.25% dextran (20 kD) overnight at 4°C. After fixation, the specimens were embedded in Embed 812 (EMS, USA) using a routine TEM embedding protocol. Ultrathin (70 nm) sections were prepared on an Ultracut S ultramicrotome (Leica, Austria) for TEM examination. After staining with uranyl acetate and lead citrate, the sections were observed using a Jeol 1400 plus TEM (Japan). Measurements of the largest diameter of mitochondria and synaptic vesicles were obtained and the average size was calculated.

### Genomic Studies

The clinical picture suggested the presence of DOORS syndrome underlying most of the alterations observed in the patient. After obtaining written informed consent from the legal guardian, genomic DNA was extracted from peripheral blood leukocytes via a standard protocol. Sanger sequencing was performed to screen for pathogenic mutations in the TBC1D24 gene, but no alterations were detected. Mutations in the ATP6V1B2 gene were assessed next, which led to the identification of the previously reported p.R506X pathogenic mutation in a heterozygous form, which is mainly known to cause DDOD syndrome (4). For apolipoprotein E (ApoE) analysis, which we considered necessary to provide context for the lack of Aβ pathology, genomic DNA was extracted from peripheral blood using a standard desalting method (12) and stored at −20°C until further use. The ApoE polymorphism was determined by polymerase chain reaction (PCR) and restriction fragment-length polymorphism. The following primers were used for amplification (13): forward primer 5′-TCC AAG GAG CTG CAG GCG CA-3′ and reverse primer 5′-ACA GAA TTC GCC CCG GCC TGG TAC ACT GCC A-3′. The amplified PCR products were digested using the HhaI restriction enzyme (Thermo Fisher Scientific, Waltham, MA, USA) and electrophoresed in a 4% agarose gel. Based on this method, the ApoE genotype of the patient was determined to be ε3/ε3.

## Discussion

Although the majority of cases of DOORS syndrome are caused by mutations in the TBC1D24 gene (2), some cases with mutations in the ATP6V1B2 gene have been identified (unpublished results). Genetic alterations in both of these genes may affect endolysosomal functioning, thus yielding a similar pathological basis for these overlapping syndromes (4, 14). However, no detailed human neuropathological assessment is available in patients with these mutations to compare the alterations with those obtained from studies of other lysosomal disorders.

Regarding the establishment of clinicopathological relationships, the pyramidal signs observed were probably related to the generalized atrophy observed on MRI, which evolved with age as a part of the syndrome, or to the presence of small lacunes, as demonstrated by macroscopic neuropathological examination; however, their prominent role was not confirmed by either MRI or microscopic neuropathological assessment. No territorial ischemic stroke or any other reason was found to underlie the slightly left-sided corticospinal symptomatology.

Drug-induced movement disorder (as a side effect of risperidone or valproate), the functional consequence of the reduced vascular reserve capacity of the basal ganglia, and a late-onset symptom of DOORS syndrome may all account for the observed Parkinsonism. If considering Parkinsonism as a drug-induced movement disorder, the presence of resting tremor before the known administration of possible initiating agents represents a major contradiction. Nevertheless, the halting of risperidone therapy (at the diagnostic admission, the patient had not received valproate) led to a remarkable improvement, with almost complete cessation of the tremor. However, subsequently, the tremor recurred and the rigidity worsened, and these signs could only be moderately controlled by low-dose levodopa treatment (100 mg t.i.d.). In line with the SPECT findings, this may support a vascular etiology; however, no prominent underlying vascular lesions were identified either by MRI or by microscopic neuropathological assessment. Regarding the etiology of the neurodegenerative presentation, no α-synuclein or tau pathology could be demonstrated by detailed neuropathological assessment of the nigrostriatal system. Therefore, a combined secondary etiology (progressively decreasing vascular reserve capacity of the basal ganglia and drug-induced aggravation of signs) is suggested as an explanation for the Parkinsonian syndrome, and its relationship with DOORS syndrome cannot be proved. Of note, Parkinsonism was reported previously to be associated with mutations in the most common causative gene for DOORS syndrome (15). In addition, individuals with TBC1D24 mutations were also noted to have dystonia and other movement disorders (16).

Seizures are hallmarks of TBC1D24-related disorders (17, 18), and the presence of seizures was also demonstrated in patients harboring the heterozygous p.E374Q or p.L398V (a large Polish family with seven affected members) mutations in the *ATP6V1B2* gene (8, 9). In these latter autosomal dominantly inherited cases with positive EEG findings, the age of onset ranged from 3 to 16 years. In our case, surprisingly, a late-onset first seizure was documented at the age of 52 years, and, in light of negative EEG findings, a question may arise regarding whether this can be designated as a specific clinical feature. However, the diagnosis of epilepsy could be established based on the presence of two unprovoked seizures occurring > 24 h apart, according to the 2014 definition provided by the International League Against Epilepsy (19), even in the absence of support from EEG, the sensitivity of which may be low regarding the detection of interictal epileptiform discharges in certain cases (20). Although primarily genetically determined epilepsy can be diagnosed, the presence of medial temporal lobe atrophy may suggest a structural origin, similar to that reported in Alzheimer's disease [AD; (21)]; however, this can also presumably be designated as a disease-related phenomenon.

There is an ongoing debate regarding whether neurodevelopmental alterations including intellectual disability and neurodegeneration, e.g., accompanied by cognitive decline, have a different pathological basis or share a common one that yields a continuum regarding symptom evolution (22). The latter hypothesis is strongly presumed in some lysosomal disorders, including NPC (22). This may also be the situation in the current case. With a pathological background of intellectual disability/cognitive decline and behavioral alterations, the examined sections revealed a complex constellation of prominent age-associated tau pathologies. We observed signs of argyrophilic grain disease (AGD), the precise significance of which in neurodegenerative disease remains debatable (23), with pretangles and astrocytic and oligodendroglial tau pathology in the limbic system. According to the stages of Saito et al. (24), this patient had stage II disease. In addition, we observed NFTs in the medial temporal lobes without apparent Aβ deposition. This pathology is now called primary age-related tauopathy [PART (25); formerly also called NFT-only dementia], which is not necessarily associated with cognitive decline. The distribution of NFTs corresponded to stage II of Braak et al. (26). Finally, we detected a prominent tau-astrogliopathy in the basal brain regions and medial temporal lobe in the frame of age-related tau-astrogliopathy [ARTAG (27)]. The regional distribution of this combined brain tau pathology is presented in Table 2 in a semi-quantitative manner.

Endolysosomal dysfunction caused by mutations in the *ATP6V1B2* gene may provide a molecular background for the present findings; however, it could not be unequivocally verified in the current case because of sample quality and availability issues resulting from an extended postmortem delay. It is well-known that lysosomal dysfunction, in particular lysosomal lipid storage disorders (including NPC disease), can initiate an NFT pathology that resembles that detected in AD and in the present case, albeit with a somewhat different regional distribution pattern [(26, 28–30); Table 2]. In the case of NPC, there is moderate involvement of basal ganglia, thalamus, and several parts of the brainstem, in addition to the regions involved in AD and in our case. Moreover, AGD without Aβ deposition was observed in cerebrotendinous xanthomatosis (CTX), a disorder that is characterized by the disturbance of cholesterol homeostasis (31). The explanation behind this phenomenon may be that lysosomal function is strongly affected by lipid homeostasis (32); therefore, altered lipid processing may cause secondary lysosomal dysfunction and vice versa. Accordingly, it was demonstrated that the distribution of tau pathology is strongly correlated with the intracellular accumulation of cholesterol in NPC (30). Based on these observations, we hypothesize that disturbance of the lysosomal system increases neurite or glial vulnerability, which then manifest as tau pathology, which is a common feature of these diseases. Of note, Aβ pathology was absent in our case with DOORS syndrome, similar to CTX and several reported cases of NPC. In fact, the few cases of NPC that have been reported to have associated Aβ pathology were exclusively ApoE ε4/ε4 homozygotes (33), which is the strongest genetic risk factor for late-onset AD. In light of these findings, the ApoE ε3/ε3 genotype of our patient appears to be consistent with the complete lack of Aβ pathology. Although the mitochondria were ultrastructurally normal, the literature reports mitochondrial dysfunction in DOORS syndrome (34, 35), tauopathies, and NPC. Interestingly, tauopathies exhibit secondary mitochondrial dysfunction (36), and reactive oxygen species promote tau modifications (37) and are upregulated in tauopathies. Mitochondrial dysfunction is also present in NPC because of lysosomal–mitochondrial contacts (38), and flies that are mutant for the TBC1D24 homolog are more sensitive to reactive oxygen species (16).

## Concluding Remarks

In conclusion, the current study was the first to present a detailed clinicopathological relationship regarding p.R506X pathogenic mutation in the *ATP6V1B2* gene. The results of the current work support the following conclusions: (1) the presence of age-associated tauopathies is demonstrated in DOORS syndrome in the forms of AGD, PART, and ARTAG, which may eventually be a consequence of lysosomal and/or mitochondrial dysfunction as discussed also in NPC, eventually in the frame of an accelerated aging process; (2) the cognitive and behavioral alterations that were present from childhood in the form of intellectual disability may be augmented by the evolution of the limbic-predominant tauopathy.

The limitation of this study was that only one case was reported; therefore, the findings need to be confirmed in additional cases. Accordingly, we propose future prospective studies using a series of patients and controls (including patients with AD and NPC) to elucidate the characteristics of endolysosomal dysfunction at different levels including a fine ultrastructural analysis and to obtain unbiased comparative quantitative measures of subregional tau pathologies.

## Ethics Statement

Written informed consent was obtained from the patient/participant's guardian/next of kin for the publication of this case report, including any identifiable data or images included within the study.

## Author Contributions

DZ, LS, PK, and LV performed the neurological workup of the patients. ZR and GK performed the neuropathological assessment of the patient. NA and PC performed the genetic analysis. RM-T performed the determination of ApoE genotype. DZ wrote the first draft of the manuscript. DZ, LS, ZR, GK, and PC wrote sections of the manuscript. All authors contributed to manuscript revision and read and approved the submitted version.

## Conflict of Interest

The authors declare that the research was conducted in the absence of any commercial or financial relationships that could be construed as a potential conflict of interest.
