# Supplementary material for: Clinicopathological Relationships in an Aged Case of DOORS Syndrome With a p.Arg506X Mutation in the ATP6V1B2 Gene
Source: Front Neurol. 2020 Aug 7;11:767. doi: 10.3389/fneur.2020.00767 (PMC7427051; doi:10.3389/fneur.2020.00767)
Supplement: Supplementary file 1 [file Data_Sheet_1.DOCX]

Supplementary Material

Clinicopathologic relationships in an aged case of DOORS syndrome with p.Arg506X mutation in the *ATP6V1B2* gene

Dénes Zádori^1*^, Levente Szalárdy^1^, Zita Reisz^2^, Gabor G. Kovacs^3,4,5^, Rita Maszlag-Török^1^, Norbert F. Ajeawung^6^, László Vécsei^1,7^, Philippe M Campeau^6,8^ and Péter Klivényi^1^

^1^Department of Neurology, Interdisciplinary Excellence Center, Faculty of Medicine, Albert Szent-Györgyi Clinical Center, University of Szeged, Szeged, Hungary

^2^Department of Pathology, Faculty of Medicine, Albert Szent-Györgyi Clinical Center, University of Szeged, Szeged, Hungary

^3^Institute of Neurology, Medical University of Vienna, Vienna, Austria

^4^Department of Laboratory Medicine and Pathobiology and Tanz Centre for Research in Neurodegenerative Disease, University of Toronto, Toronto, Ontario, Canada

^5^Laboratory Medicine Program & Krembil Brain Institute, University Health Network, Toronto, Ontario, Canada

^6^CHU Sainte-Justine Research Center, Université de Montréal, Montréal, Quebec, Canada.

^7^MTA-SZTE Neuroscience Research Group, University of Szeged, Szeged, Hungary

^8^Department of Pediatrics, Sainte-Justine University Hospital Center, Montreal, Quebec, Canada

*** Correspondence:**Dénes Zádori MD, PhD
Department of Neurology, Faculty of Medicine, Albert Szent-Györgyi Clinical Center, University of Szeged, Szeged, Hungary
H-6725 Szeged, Semmelweis u. 6.
E-mail: zadori.denes@med.u-szeged.hu
Phone: +36-62-545-348

# Supplementary Material 1

The video demonstrating the neurological assessment of patient II-2. FigShare link: <https://figshare.com/s/0709ffb81185e86b1009>

# Supplementary Material 2


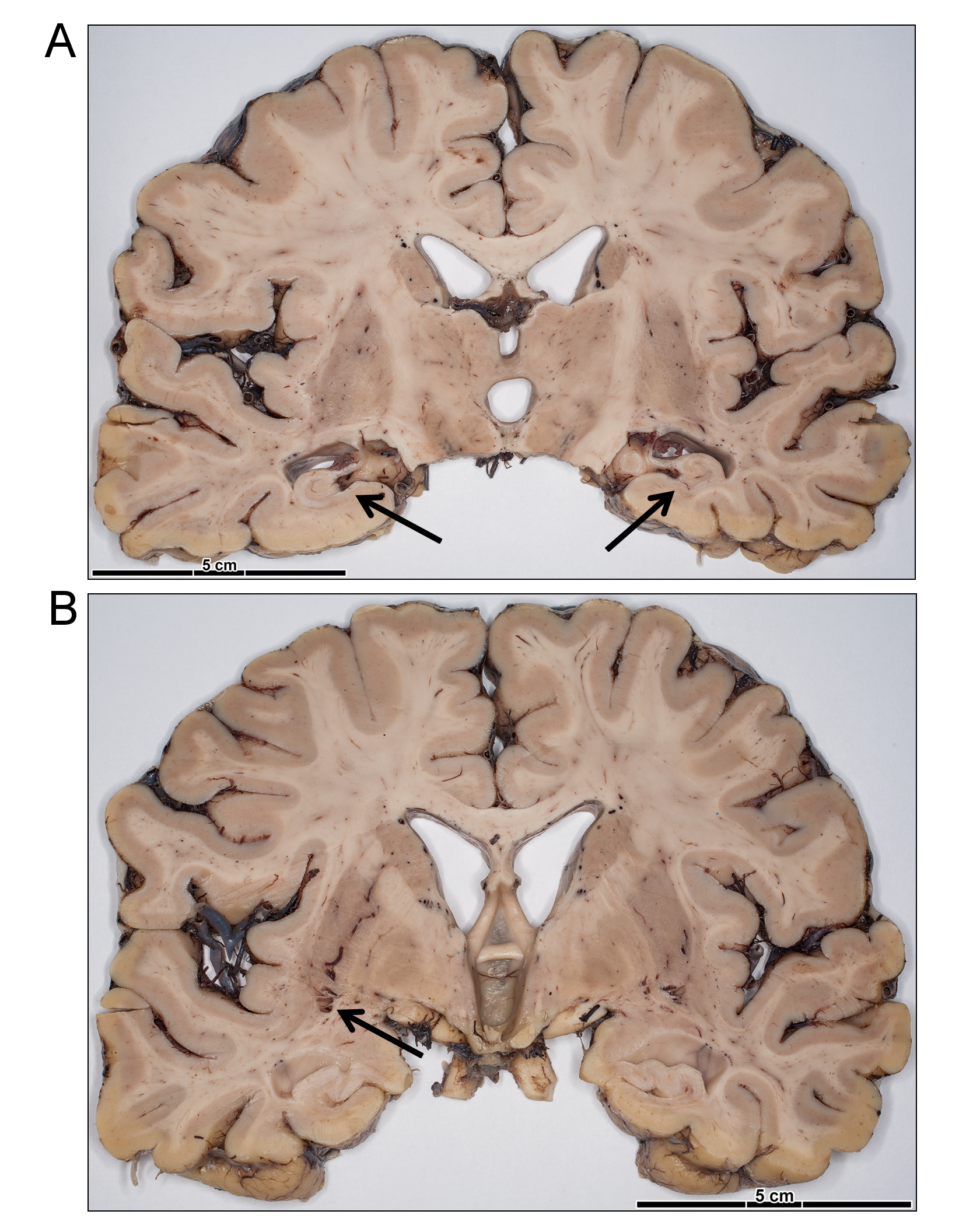


Macroscopic neuropathological alterations of an aged patient with DOORS syndrome. On the coronal sections the mild thinning of the temporal gyri were accompanied by moderate symmetrical, bilateral hippocampal atrophy (black arrows; (**A**)). Small lacunes were detected both in the putamen and in the surrounding deep white matter, whereas the caudate and thalamic nuclei were unaffected (black arrow (**B**)).

# Supplementary Material 3


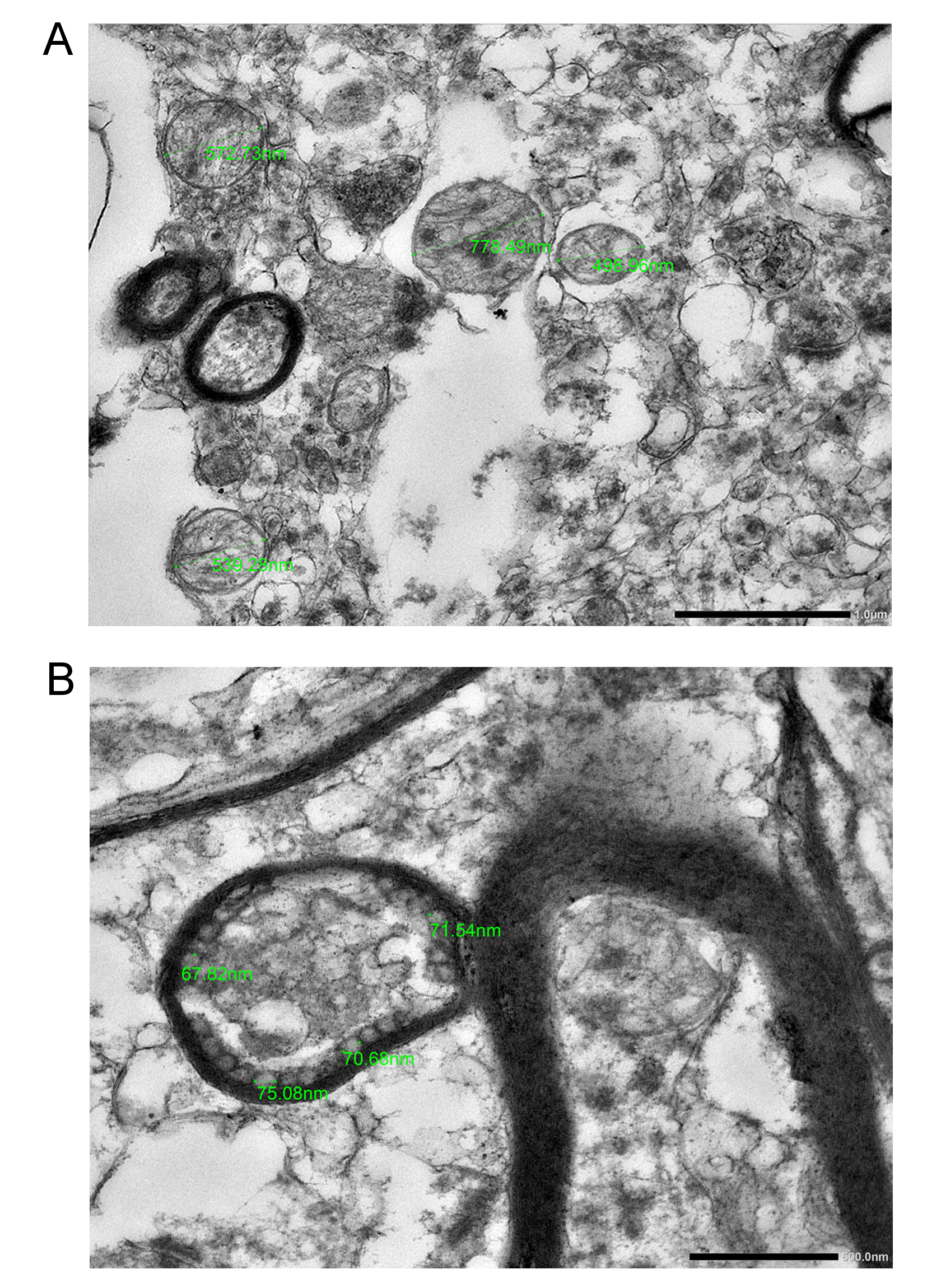


Electron microscopy targeting the visualization of mitochondria and synaptic vesicles. The diameter of mitochondria (A) and synaptic vesicles (B) were measured to be within normal ranges.
